# Supplementary material for: Habitat Imaging Biomarkers for Diagnosis and Prognosis in Cancer Patients Infected with COVID-19
Source: Cancers (Basel). 2022 Dec 31;15(1):275. doi: 10.3390/cancers15010275 (PMC9818576; doi:10.3390/cancers15010275)
Supplement: Supplementary file 1 [file cancers-15-00275-s001.zip › D3CODE members.pdf]

**Supplementary Materials**

**D3CODE Team Authors List**

- Ashley Aaroe, MD
- Thomas A. Aloia, MD
- Lee Andrews II
- Kiran K. Badami, BS
- Janna A. Baganz, MA
- Pratibha Bajwa, MS
- Lori R. Baker, BA
- Gregory R. Barbosa, MBA
- Hannah C. Beird, BS PhD
- Matt Bourgeois
- Kristy Brock, PhD
- Elizabeth M. Burton, MBA
- Juan Cata, MD
- Caroline Chung, MD
- Michael Cutherell
- John A. Cuenca, MD
- Pierre B. Cyr, MA
- Bouthaina Dabaja, MD
- Hiba Dagher, MD
- Kevin M. Daniels, BA
- Mary Domask, BS
- Giulio Draetta, MD PhD
- Sarah Fisher, MD
- Katy Elizabeth French, MD
- Andrew Futreal, PhD
- Maria Gaeta, MD
- Christopher Gibbons, PhD
- Myrna Godoy, MD
- Drew Goldstein
- Jillian Gunther, MD PhD
- Cristhiam Hernandez, MD
- Kate Hutcheson, PhD
- David Jaffray, PhD
- Jeff Jin, PhD
- Teny Matthew John, MBBS
- Trey Kell
- Mark Knafl, MS
- Anai Kothari, MD MS
- Rayson C. Kwan, BBA
- J Jack Lee, PhD
- Yue Liao, PhD

- Jennifer Litton, MD
- Alex Liu, MBA
- Kevin W. McEnery, MD
- Mary McGuire, PhD
- Benjamin Mescher
- Tego Musunuru, MD
- Mayoora Muthu,DO
- Joseph L. Nates, MD
- Craig S. Owen, BBa
- Priyadharshini Padmakumar, BS
- Melody Page
- Nicholas Palaskas, MD
- Jay J. Patel, MCS
- Sabitha Prabhakaran, PhD
- Lucas Ramsey
- Vinod Ravi, MD
- Ludivine Russell, MS
- Bilja Sajith, PhD
- Paul A. Scheet, PhD
- Stephanie Schmidt, PhD
- Kenna R. Shaw, PhD
- Sanjay Shete, PhD
- Daniel P. Shoenthal, MD
- Lessley J. Stoltenberg, BS
- Ishwaria Subbiah, MD
- Chuck Suitor, MS
- Hussein Tawbi, MD, PhD
- Phillip Thompson
- Anastasia Turin, BS
- Samir Unni
- Benju Vicknamparampil, BS
- Max C. Weber, MBA
- John Weinstein, MD PhD
- Zoe Williams
- Scott E. Woodman, MD PhD
- Mark C. Wozny, PhD
- Carol Wu, MD
- Jia Wu, PhD
- James C. Yao, MD
- Chingyi Young, MS
- Emily Yu
- Steven Zatorski
