# Supplementary material for: Habitat Imaging Biomarkers for Diagnosis and Prognosis in Cancer Patients Infected with COVID-19
Source: Cancers (Basel). 2022 Dec 31;15(1):275. doi: 10.3390/cancers15010275 (PMC9818576; doi:10.3390/cancers15010275)
Supplement: Supplementary file 1 [file cancers-15-00275-s001.zip › Supplement Table S1.pdf]

Table S1. Comparison of our proposed COVID-19 diagnosis approach with other state-of-the-art techniques.

Data type	Study	Sample size	Accuracy	AUC
CT	Zhao et al [1]	112	0.9460	0.9470
CT	Javaheri et al [2]	335	0.9333	0.9400
Clinical variables	Zhou et al [3]	411	0.8700	0.8900
Cough sounds	Melek [4]	180	0.9833	0.9860
Symptoms + sensor data	Quer et al [5]	30529	-	0.8000
	(1)	951	1.0000	1.0000

1. Zhao, C.; Xu, Y.; He, Z.; Tang, J.; Zhang, Y.; Han, J.; Shi, Y.; Zhou, W. Lung segmentation and automatic detection of COVID-19 using radiomic features from chest CT images. *Pattern Recognition* **2021**, *119*, 108071.
2. Javaheri, T.; Homayounfar, M.; Amoozgar, Z.; Reiazi, R.; Homayounieh, F.; Abbas, E.; Laali, A.; Radmard, A.R.; Gharib, M.H.; Mousavi, S.A.J. CovidCTNet: an open-source deep learning approach to diagnose covid-19 using small cohort of CT images. *NPJ digital medicine* **2021**, *4*, 1-10.
3. Zhou, J.; Lee, S.; Wang, X.; Li, Y.; Wu, W.K.K.; Liu, T.; Cao, Z.; Zeng, D.D.; Leung, K.S.K.; Wai, A.K.C. Development of a multivariable prediction model for severe COVID-19 disease: a population-based study from Hong Kong. *NPJ digital medicine* **2021**, *4*, 1-9.
4. Melek, M. Diagnosis of COVID-19 and non-COVID-19 patients by classifying only a single cough sound. *Neural Computing and Applications* **2021**, *33*, 17621-17632.
5. Quer, G.; Radin, J.M.; Gadaleta, M.; Baca-Motes, K.; Ariniello, L.; Ramos, E.; Kheterpal, V.; Topol, E.J.; Steinhubl, S.R. Wearable sensor data and self-reported symptoms for COVID-19 detection. *Nature Medicine* **2021**, *27*, 73-77.
