# Supplementary material for: Habitat Imaging Biomarkers for Diagnosis and Prognosis in Cancer Patients Infected with COVID-19
Source: Cancers (Basel). 2022 Dec 31;15(1):275. doi: 10.3390/cancers15010275 (PMC9818576; doi:10.3390/cancers15010275)
Supplement: Supplementary file 1 [file cancers-15-00275-s001.zip › Supplement Table S2.pdf]

Table S2. Performance comparison of the different models for Admission prediction

**Methods**	**Cohort**							
	General				Cancer			
	Acc	Sen	Spe	AUC	Acc	Sen	Spe	AUC
**LR**	0.9903	1.0000	0.9808	1.0000	0.8580	0.9338	0.8069	0.9494
**RF**	0.9748	1.0000	0.9517	1.0000	0.8550	0.9835	0.7834	0.9493
**SVM**	0.9709	1.0000	0.9446	1.0000	0.9615	0.9636	0.9595	0.9842
**GAM**	0.9845	1.0000	0.9697	1.0000	0.8609	0.9837	0.7907	0.9608
