# Supplementary material for: Habitat Imaging Biomarkers for Diagnosis and Prognosis in Cancer Patients Infected with COVID-19
Source: Cancers (Basel). 2022 Dec 31;15(1):275. doi: 10.3390/cancers15010275 (PMC9818576; doi:10.3390/cancers15010275)
Supplement: Supplementary file 1 [file cancers-15-00275-s001.zip › Supplement Table S3.pdf]

Table S3. Performance comparison of the different models for ICU prediction.

| Methods | Cohort | | | | | | | |
|---|---|---|---|---|---|---|---|---|
| | General | | | | Cancer | | | |
| | Acc | Sen | Spe | AUC | Acc | Sen | Spe | AUC |
| **LR** | 0.8883 | 0.8654 | 0.9200 | 0.9623 | 0.9928 | 1.0000 | 0.9859 | 1.0000 |
| **RF** | 0.8771 | 0.9625 | 0.8081 | 0.9717 | 0.9356 | 1.0000 | 0.8861 | 1.0000 |
| **SVM** | 0.9609 | 0.9406 | 0.9872 | 0.9758 | 0.9928 | 1.0000 | 0.9859 | 1.0000 |
| **GAM** | 0.8911 | 0.9873 | 0.8159 | 0.9765 | 0.9427 | 1.0000 | 0.8974 | 1.0000 |
