# Supplementary material for: Habitat Imaging Biomarkers for Diagnosis and Prognosis in Cancer Patients Infected with COVID-19
Source: Cancers (Basel). 2022 Dec 31;15(1):275. doi: 10.3390/cancers15010275 (PMC9818576; doi:10.3390/cancers15010275)
Supplement: Supplementary file 1 [file cancers-15-00275-s001.zip › Supplement Table S4.pdf]

Table S4. Performance comparison of the different classification models for COVID-19 diagnosis using deep features extracted from the general and cancer cohorts. Acc: accuracy; Sen: sensitivity; Spe: specificity; AUC: area under the receiver operating characteristic curve

Methods	Cohort							
	General				Cancer			
	Acc	Sen	Spe	AUC	Acc	Sen	Spe	AUC
**LR**	0.9379	0.8559	0.9613	0.9792	0.9571	0.9932	0.9380	0.9737
**RF**	0.9397	0.8455	0.9681	0.9779	0.9571	0.9675	0.9511	0.9750
**SVM**	0.9360	0.8268	0.9703	0.9788	0.9571	0.9932	0.9380	0.9702
**GAM**	0.9379	0.8684	0.9568	0.9738	0.9571	0.9675	0.9511	0.9698
