# Supplementary material for: Habitat Imaging Biomarkers for Diagnosis and Prognosis in Cancer Patients Infected with COVID-19
Source: Cancers (Basel). 2022 Dec 31;15(1):275. doi: 10.3390/cancers15010275 (PMC9818576; doi:10.3390/cancers15010275)
Supplement: Supplementary file 1 [file cancers-15-00275-s001.zip › Supplement Table S5.pdf]

Table S5. Performance of the COVID-19 diagnostic models trained with deep features extracted on the general cohort and applied on the cancer cohort. Acc: accuracy; Sen: sensitivity; Spe: specificity; AUC: area under the receiver operating characteristic curve

**Methods**	**All**			
	Acc	Sen	Spe	AUC
**LR**	0.8714	1.0000	0.8269	0.9652
**RF**	0.8810	1.0000	0.8377	0.9633
**SVM**	0.9143	1.0000	0.8776	0.9673
**GAM**	0.8429	1.0000	0.7963	0.9648
