# Supplementary material for: Habitat Imaging Biomarkers for Diagnosis and Prognosis in Cancer Patients Infected with COVID-19
Source: Cancers (Basel). 2022 Dec 31;15(1):275. doi: 10.3390/cancers15010275 (PMC9818576; doi:10.3390/cancers15010275)
Supplement: Supplementary file 1 [file cancers-15-00275-s001.zip › Supplement Table S6.pdf]

Table S6. Performance comparison of the different classification models for admission prediction using deep features extracted from the general and cancer cohorts. Acc: accuracy; Sen: sensitivity; Spe: specificity; AUC: area under the receiver operating characteristic curve

**Methods**	**Cohort**							
	General				Cancer			
	Acc	Sen	Spe	AUC	Acc	Sen	Spe	AUC
**LR**	0.9922	1.0000	0.9846	1.0000	0.8817	0.9701	0.8235	0.9565
**RF**	0.9728	1.0000	0.9481	1.0000	0.8728	1	0.8000	0.9600
**SVM**	0.9942	1.0000	0.9884	1.0000	0.9675	0.9532	0.9820	0.9924
**GAM**	0.9883	1.0000	0.9771	1.0000	0.8846	0.9847	0.8213	0.9742
