# Supplementary material for: Habitat Imaging Biomarkers for Diagnosis and Prognosis in Cancer Patients Infected with COVID-19
Source: Cancers (Basel). 2022 Dec 31;15(1):275. doi: 10.3390/cancers15010275 (PMC9818576; doi:10.3390/cancers15010275)
Supplement: Supplementary file 1 [file cancers-15-00275-s001.zip › Supplement Table S7.pdf]

Table S7. Performance comparison of the different classification models for ventilation prediction using deep features extracted from the general and cancer cohorts. Acc: accuracy; Sen: sensitivity; Spe: specificity; AUC: area under the receiver operating characteristic curve

**Methods**	**Cohort**							
	General				Cancer			
	Acc	Sen	Spe	AUC	Acc	Sen	Spe	AUC
**LR**	0.9305	0.9888	0.8866	0.9942	0.9889	1.0000	0.9778	1.0000
**RF**	0.9113	1.0000	0.8520	0.9995	0.9735	1.0000	0.9483	1.0000
**SVM**	0.9952	1.0000	0.9907	1.0000	0.9602	1.0000	0.9244	1.0000
**GAM**	0.9376	1.0000	0.8912	1.0000	0.9845	1.0000	0.9692	1.0000
