# Supplementary material for: Habitat Imaging Biomarkers for Diagnosis and Prognosis in Cancer Patients Infected with COVID-19
Source: Cancers (Basel). 2022 Dec 31;15(1):275. doi: 10.3390/cancers15010275 (PMC9818576; doi:10.3390/cancers15010275)
Supplement: Supplementary file 1 [file cancers-15-00275-s001.zip › Supplement Table S8.pdf]

Table S8. Performance comparison of the different classification models for ICU prediction using deep features extracted from the general and cancer cohorts. Acc: accuracy; Sen: sensitivity; Spe: specificity; AUC: area under the receiver operating characteristic curve

Methods	Cohort							
	General				Cancer			
	Acc	Sen	Spe	AUC	Acc	Sen	Spe	AUC
**LR**	0.9379	0.8559	0.9613	0.9792	0.9571	0.9932	0.9380	0.9737
**RF**	0.9397	0.8455	0.9681	0.9779	0.9571	0.9675	0.9511	0.9750
**SVM**	0.9360	0.8268	0.9703	0.9788	0.9571	0.9932	0.9380	0.9702
**GAM**	0.9379	0.8684	0.9568	0.9738	0.9571	0.9675	0.9511	0.9698

Supplement Table 5. Performance of the COVID-19 diagnostic models trained with deep features extracted on the general cohort and applied on the cancer cohort. Acc: accuracy; Sen: sensitivity; Spe: specificity; AUC: area under the receiver operating characteristic curve

**Methods**	**All**			
	Acc	Sen	Spe	AUC
**LR**	0.8714	1.0000	0.8269	0.9652
**RF**	0.8810	1.0000	0.8377	0.9633
**SVM**	0.9143	1.0000	0.8776	0.9673
**GAM**	0.8429	1.0000	0.7963	0.9648

Supplement Table 6. Performance comparison of the different classification models for admission prediction using deep features extracted from the general and cancer cohorts. Acc: accuracy; Sen: sensitivity; Spe: specificity; AUC: area under the receiver operating characteristic curve

**Methods**	**Cohort**							
	General				Cancer			
	Acc	Sen	Spe	AUC	Acc	Sen	Spe	AUC
**LR**	0.9922	1.0000	0.9846	1.0000	0.8817	0.9701	0.8235	0.9565
**RF**	0.9728	1.0000	0.9481	1.0000	0.8728	1	0.8000	0.9600
**SVM**	0.9942	1.0000	0.9884	1.0000	0.9675	0.9532	0.9820	0.9924
**GAM**	0.9883	1.0000	0.9771	1.0000	0.8846	0.9847	0.8213	0.9742

Supplement Table 7. Performance comparison of the different classification models for ventilation prediction using deep features extracted from the general and cancer cohorts. Acc: accuracy; Sen: sensitivity; Spe: specificity; AUC: area under the receiver operating characteristic curve

**Methods**	**Cohort**							
	General				Cancer			
	Acc	Sen	Spe	AUC	Acc	Sen	Spe	AUC
**LR**	0.9305	0.9888	0.8866	0.9942	0.9889	1.0000	0.9778	1.0000
**RF**	0.9113	1.0000	0.8520	0.9995	0.9735	1.0000	0.9483	1.0000
**SVM**	0.9952	1.0000	0.9907	1.0000	0.9602	1.0000	0.9244	1.0000
**GAM**	0.9376	1.0000	0.8912	1.0000	0.9845	1.0000	0.9692	1.0000

Supplement Table 8. Performance comparison of the different classification models for ICU prediction using deep features extracted from the general and cancer cohorts. Acc: accuracy; Sen: sensitivity; Spe: specificity; AUC: area under the receiver operating characteristic curve

Methods	Cohort							
	General				Cancer			
	Acc	Sen	Spe	AUC	Acc	Sen	Spe	AUC
**LR**	0.8939	0.8738	0.9211	0.9668	0.9905	1.0000	0.9813	1.0000
**RF**	0.8771	0.9868	0.7961	0.9763	0.9547	1.0000	0.9170	1.0000
**SVM**	0.9609	0.9450	0.9810	0.9842	0.9547	1.0000	0.9170	1.0000
**GAM**	0.8827	0.9870	0.8039	0.9864	0.9475	1.0000	0.9052	1.0000
