# Supplementary figures and images for: Habitat Imaging Biomarkers for Diagnosis and Prognosis in Cancer Patients Infected with COVID-19
Source: Cancers (Basel). 2022 Dec 31;15(1):275. doi: 10.3390/cancers15010275 (PMC9818576; doi:10.3390/cancers15010275)

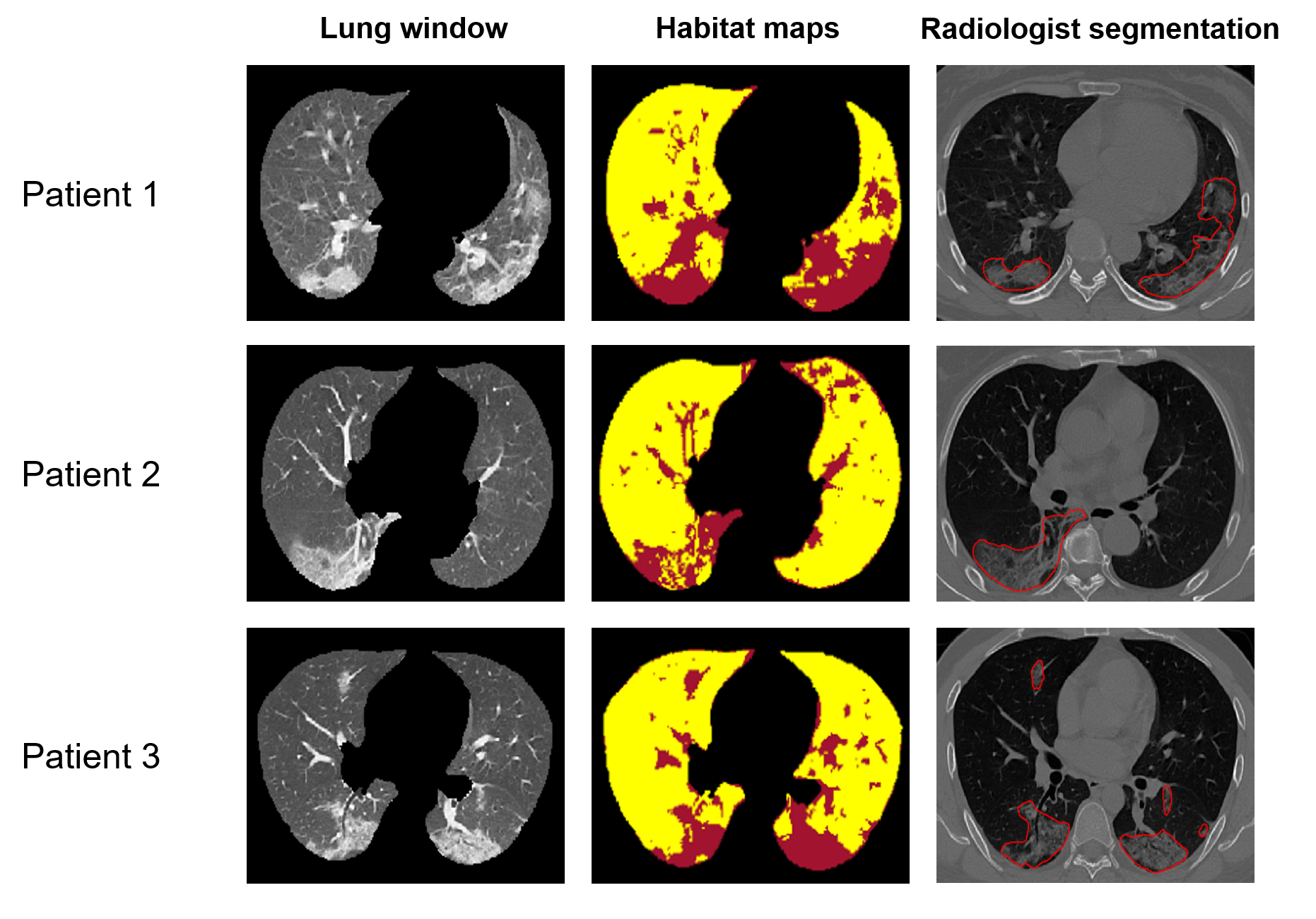

Supplement: Supplementary file 1 [file cancers-15-00275-s001.zip › Supplement Figure S1.tif]

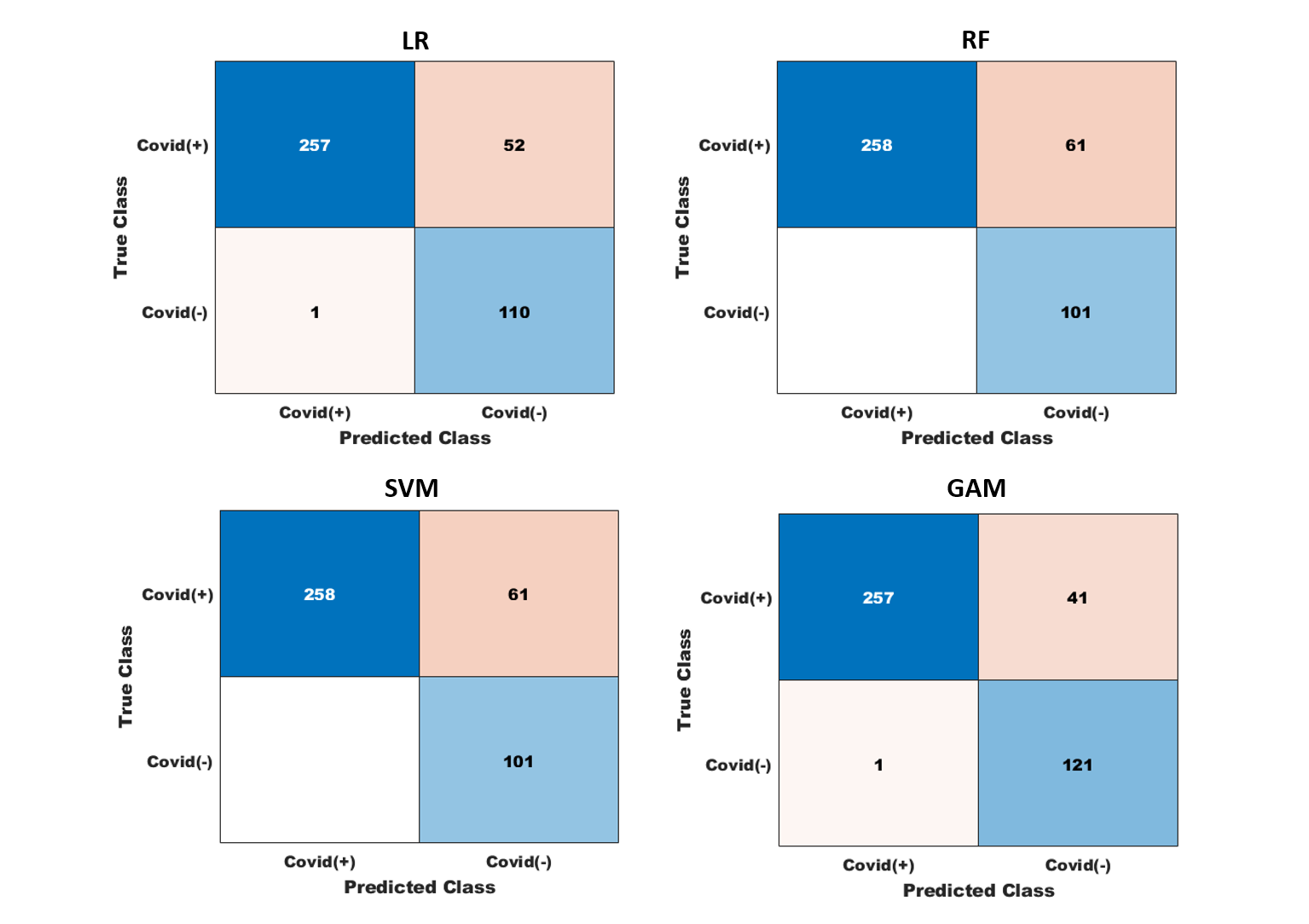

Supplement: Supplementary file 1 [file cancers-15-00275-s001.zip › Supplement Figure S2.tif]

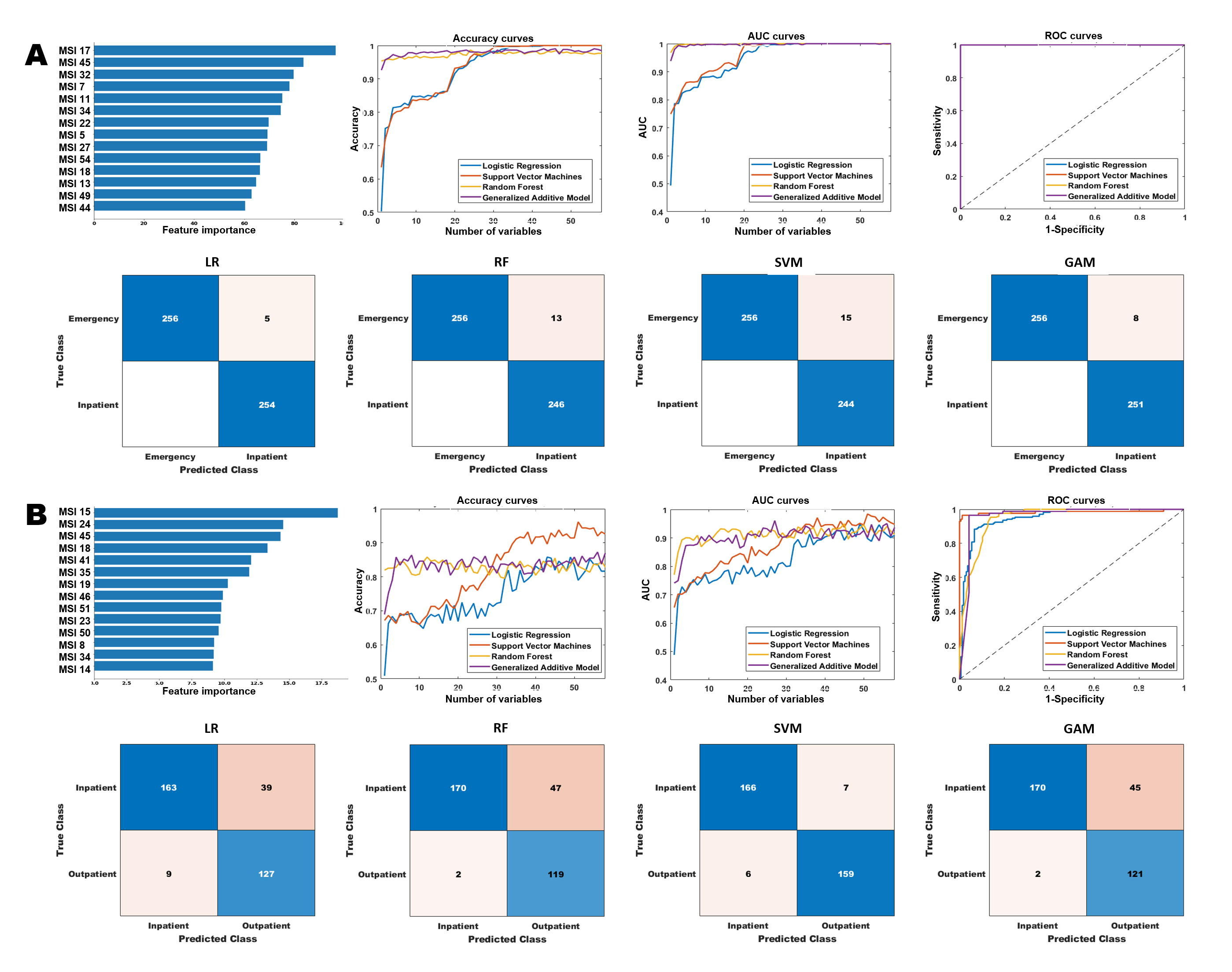

Supplement: Supplementary file 1 [file cancers-15-00275-s001.zip › Supplement Figure S3.tif]

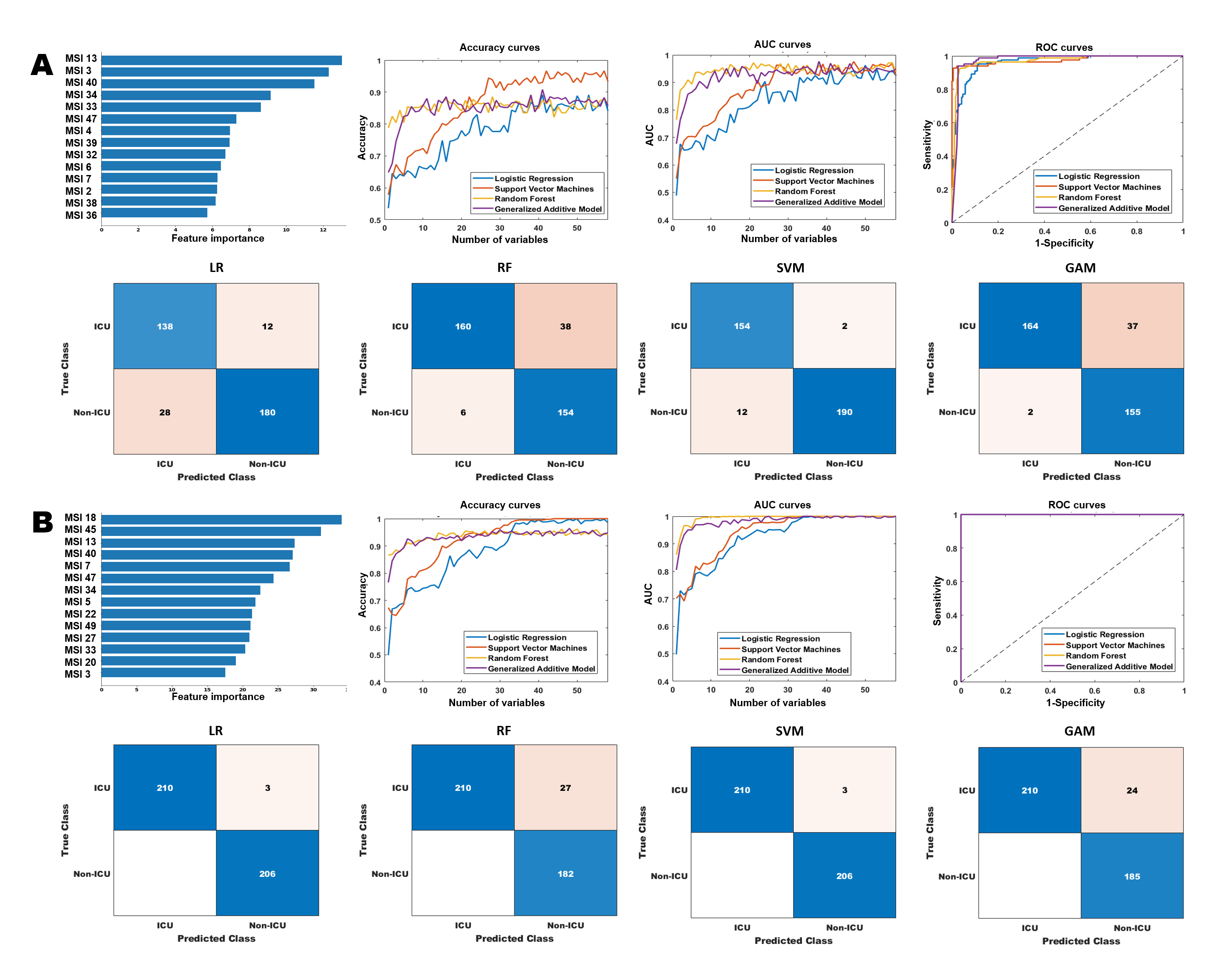

Supplement: Supplementary file 1 [file cancers-15-00275-s001.zip › Supplement Figure S4.tif]

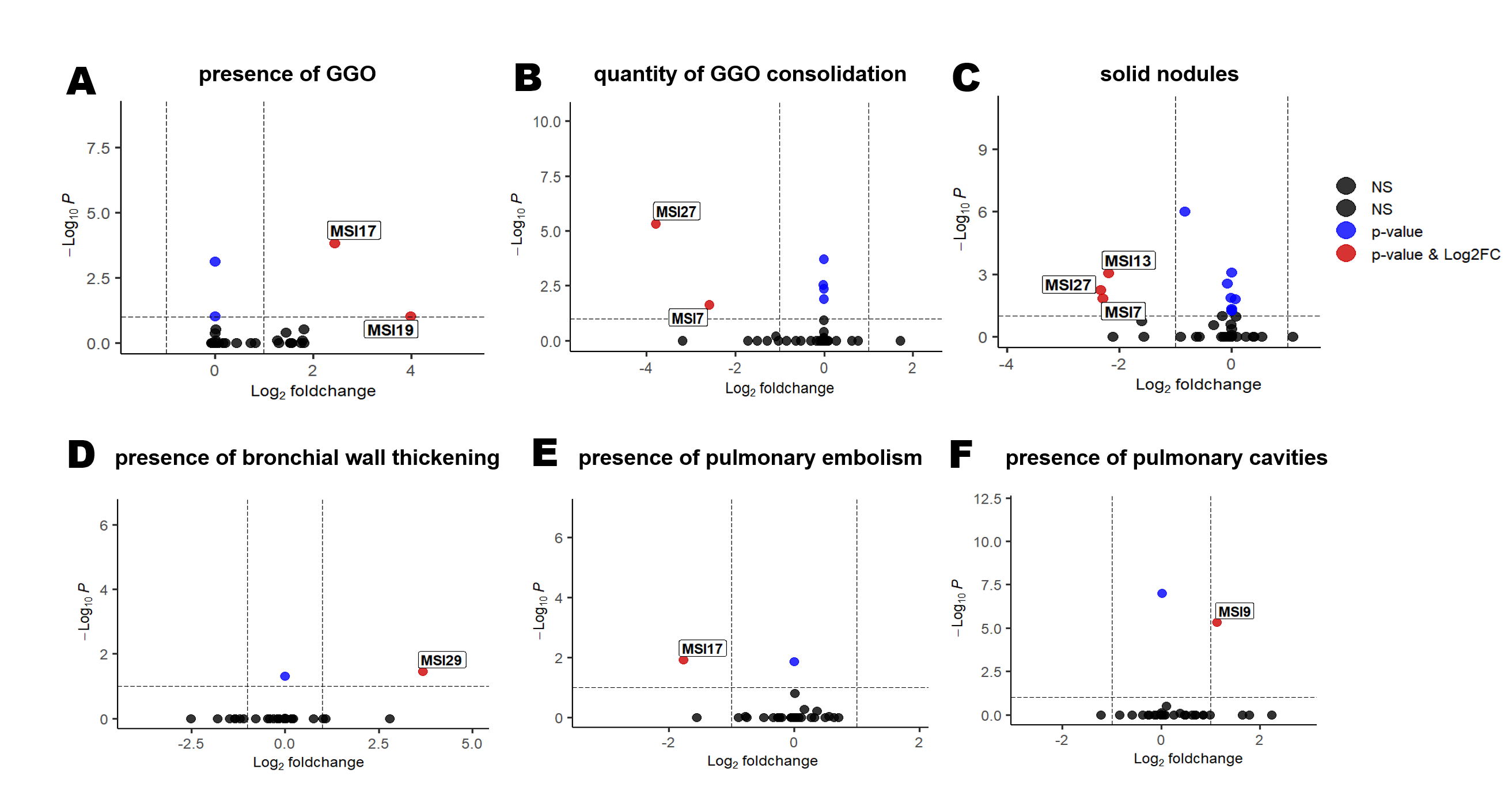

Supplement: Supplementary file 1 [file cancers-15-00275-s001.zip › Supplement Figure S5.tif]
